# Comparison between Prehospital Mechanical Cardiopulmonary Resuscitation (CPR) Devices and Manual CPR for Out-of-Hospital Cardiac Arrest: A Systematic Review, Meta-Analysis, and Trial Sequential Analysis

**DOI:** 10.3390/jcm11051448

**Published:** 2022-03-07

**Authors:** Cheng-Ying Chiang, Ket-Cheong Lim, Pei Chun Lai, Tou-Yuan Tsai, Yen Ta Huang, Ming-Jen Tsai

**Affiliations:** 1 Department of Emergency Medicine, Ditmanson Medical Foundation Chia-Yi Christian Hospital, Chiayi City 600, Taiwan; chvv7552@gmail.com (C.-Y.C.); limkc20@gmail.com (K.-C.L.); 2 Education Center, National Cheng Kung University Hospital, College of Medicine, National Cheng Kung University, Tainan 704, Taiwan; debbie0613.lai@gmail.com; 3 School of Medicine, Tzu Chi University, Hualien 970, Taiwan; 96311123@gms.tcu.edu.tw; 4 Department of Emergency Medicine, Dalin Tzu Chi Hospital, Buddhist Tzu Chi Medical Foundation, Chiayi City 622, Taiwan; 5 Department of Surgery, National Cheng Kung University Hospital, College of Medicine, National Cheng Kung University, Tainan 704, Taiwan

**Keywords:** cardiac arrest, resuscitation, cardiopulmonary resuscitation, mechanical, pre-hospital, out of hospital cardiac arrest

## Abstract

In pre-hospital settings, efficient cardiopulmonary resuscitation (CPR) is challenging; therefore, the application of mechanical CPR devices continues to increase. However, the evidence of the benefits of using mechanical CPR devices in pre-hospital settings for adult out-of-hospital cardiac arrest (OHCA) is controversial. This meta-analysis compared the effects of mechanical and manual CPR applied in the pre-hospital stage on clinical outcomes after OHCA. Cochrane Library, PubMed, Embase, and ClinicalTrials.gov were searched from inception until October 2021. Studies comparing mechanical and manual CPR applied in the pre-hospital stage for survival outcomes of adult OHCA were eligible. Data abstraction, quality assessment, meta-analysis, trial sequential analysis (TSA), and grading of recommendations, assessment, development, and evaluation were conducted. Seven randomized controlled and 15 observational studies were included. Compared to manual CPR, pre-hospital use of mechanical CPR showed a positive effect in achieving return of spontaneous circulation (ROSC) and survival to admission. No difference was found in survival to discharge and discharge with favorable neurological status, with inconclusive results in TSA. In conclusion, pre-hospital use of mechanical CPR devices may benefit adult OHCA in achieving ROSC and survival to admission. With low certainty of evidence, more well-designed large-scale randomized controlled trials are needed to validate these findings.

## 1. Introduction

Out-of-hospital cardiac arrest (OHCA) is a universal concern [1]. The global incidence is approximately 30–97 individuals per 100,000 person-years [2,3]. Although the management of OHCA has progressed, the survival rate remains poor, around 3.1% to 20.4% across the world [2,4]. Achieving survival from OHCA relies on implementing the integral chain of survival [5]. It includes early activation of the emergency medical services (EMS) system, provision of high-quality cardiopulmonary resuscitation (CPR), early defibrillation, advanced resuscitation, post-cardiac arrest care, and recovery [5]. Early administration of high-quality CPR plays an important role in achieving return of spontaneous circulation (ROSC) and preserving brain perfusion following ROSC [5]. However, efficient CPR in pre-hospital settings is challenging, especially when moving patients and during ambulance transport. The safety of EMS crews is also an issue when performing CPR, such as in a moving ambulance or resuscitating patients with coronavirus disease 2019 (COVID-19). Hence, the use of mechanical CPR devices in prehospital settings continues to increase and is recommended by professional societies in resuscitating COVID-19 patients [6,7].

However, the effect of mechanical CPR devices on the clinical outcomes of OHCA remains controversial and lacks evidence regarding the benefits of mechanical CPR for OHCA patients compared to manual CPR [8,9,10,11,12]. Zhu et al. conducted a meta-analysis in 2019, including nine randomized controlled trials (RCTs) and six non-RCTs, and found no significant differences in the resuscitative effects between mechanical and manual CPR in OHCA patients [8]. Similar result was found in Liu et al. in 2019 comparing manual CPR and mechanical CPR with the Lund University Cardiac Assist System (LUCAS) device [11]. Previous meta-analyses pooled the studies with “in-hospital” and “pre-hospital” use of mechanical CPR devices and concluded that mechanical CPR is not superior to manual CPR for OHCA. However, the resources in in-hospital settings are likely to be better than those in the pre-hospital stage, e.g., more personnel for maintaining the good quality of CPR, and a more spacious environment, and better equipment and medication. Hence, it is reasonable that studies investigating the “in-hospital” use of mechanical CPR for OHCA patients did not show benefits compared to manual CPR [13,14,15,16]. A further issue is whether previous meta-analyses had sufficient statistical power. Recently, increasing evidence including large-scale cohort and RCTs has shown the benefit of prehospital use of mechanical CPR devices [17,18,19]. Hence, a new Systemic Review and Meta-Analysis (SRMA) is needed to analyze the benefit between the use of manual CPR and mechanical CPR applied specifically in a “pre-hospital” setting. The aim of this study was to conduct a systematic review, meta-analysis, and trial sequential analysis of the published literature on the “prehospital” use of mechanical CPR devices compared to manual CPR for adult OHCA.

## 2. Materials and Methods

This SRMA was conducted according to the latest statement of the Preferred Reporting Items for Systemic Reviews and Meta-Analysis (PRISMA) [20]. Our protocol was registered on PROSPERO (CRD42021286570).

### 2.1. Inclusion Criteria

Studies were included if the participants were adult patients with OHCA, the intervention was the use of an automated mechanical CPR device in the prehospital stage (including at the scene of cardiac arrest or during ambulance transport), the comparison was with manual CPR, and the outcome indicators were survival-related outcomes. Primary outcomes were rates of ROSC and survival to hospital admission, which most directly reflect the effect and quality of CPR performed in the prehospital stage. The secondary outcomes, such as survival to discharge or 30 days, and survival to discharge with favorable neurological status (defined as Cerebral Performance Category: 1–2, Modified Rankin Scale: 0–2, or Glasgow coma scale ≥ 13), were likely influenced by post-resuscitation care. Any study with at least one of the aforementioned outcome measurements was included, comprising RCTs and non-RCTs.

### 2.2. Exclusion Criteria

Excluded studies: Studies that recruited patients with in-hospital cardiac arrest (IHCA) or OHCA who received mechanical CPR after arriving at the emergency department (ED) but not in the pre-hospital stage; studies including OHCA younger than 18 years, animal studies, simulation studies, or cardiac arrest caused by hypothermia, drowning, trauma, and toxic substances with a unique pathophysiology; studies using non-automated mechanical CPR devices, such as non-powered active devices; studies evaluating the harm, cost-effectiveness, or user ability as outcomes; and studies that were not the full-length article or without detailed description in the methodology. Articles with related studies, such as subgroup analysis that were published from the same institutions or individuals were excluded, but the most comprehensive one was retained. We also excluded studies designed to cross-over the implementation of manual CPR and mechanical CPR in individual participants.

### 2.3. Search Methods

We searched the PubMed, Embase, Cochrane Library, and ClinicalTrials.gov with the keywords of “cardiac arrest,” “heart arrest,” “cardiopulmonary resuscitation,” “CPR,” “chest compression,” “mechanical,” “Lucas,” “Autopulse,” and “Load distributing band” from inception until 27 October 2021. No language restrictions were imposed. The detailed search strategy is presented in Supplementary Table S1. We reviewed the references of eligible papers, similar articles recommended by the PubMed algorithm, and published systematic reviews to identify candidate trials that were not listed in the original database.

### 2.4. Selection of Studies

Two investigators independently screened the titles and abstracts of the studies identified from the database searches. We obtained the full-text articles for the review for more thorough screening and eligibility assessment using the same inclusion and exclusion criteria. Disagreements were resolved through consensus, and a third reviewer was involved if there was no agreement.

### 2.5. Data Extraction

Two investigators extracted data in an independent, consistent fashion using a preformed format. Data extraction included the name of the first author, year of publication, country where the study was conducted, study design, sample size, type of mechanical CPR device, total number of participants per treatment arm, and number of participants achieving the set primary and secondary outcomes. For RCTs, the data from the intention-to-treat analysis were chosen for data extraction. For non-RCTs, matched case-control data were extracted, if applicable.

### 2.6. Literature Quality Evaluation

Two authors independently assessed the risk of bias (RoB) for included studies by using a revised Cochrane risk of bias tool for randomized trials (RoB 2.0) for RCTs [21] and risk of bias in non-randomized studies of interventions (ROBINS-I) tool for non-RCTs [22].

### 2.7. Statistical Analysis

We performed a meta-analysis of the data using Review Manager version 5.41 (Cochrane Collaboration). A random-effects model was used to calculate summary statistics due to anticipated heterogeneity. Forest plots with odds ratios (ORs) and 95% confidence intervals (CIs) were analyzed using the Mantel-Haenszel (M-H) method for dichotomous data. Statistical significance was set at *p* < 0.05. Heterogeneity among studies was measured using *I*^2^ statistics. If *I*^2^ was higher than 50%, substantial heterogeneity was indicated. Sensitivity analyses of fixed-effect model, different study designs, studies with low RoB and subgroup analyses of different types of mechanical CPR devices and geographic locations where the study was conducted were performed.

### 2.8. Trial Sequential Analysis

Trial sequential analysis (TSA) was applied to quantify the statistical reliability of data by repetitive and cumulative testing for meta-analyses [23]. We conducted this analysis using TSA software version 0.9.5.10 Beta (Copenhagen Trial Unit, Center for Clinical Intervention Research, Copenhagen, Denmark). We applied a two-sided test, set Type I error of 5% and power of 80% and assumed a 10% relative risk reduction for mechanical CPR. The O’Brien–Fleming monitoring boundaries were applied for hypothesis testing, and a random-effects model with the Biggerstaff-Tweedie method was used [24]. The incidence of the control arm was filled in the “overall events/total cases” of the measured outcome in the manual CPR group of the enrolled studies. A Z-curve was constructed using cumulative evidence of trials over time. Either the Z-curve crossed the O’Brien-Fleming boundaries before the estimated required information size (RIS) was reached or the Z-curve was higher than 1.96 when the accumulated size was larger than RIS were considered true positives. Otherwise, a true negative was considered if the Z-curve entered the futility area. A total sample size that did not reach the RIS was defined as underpower.

### 2.9. Grading of the Certainty of Evidence

The quality of the overall certainty of evidence (CoE) was assessed using the Grading of Recommendations Assessment, Development, and Evaluation (GRADE) methodology for each outcome [25]. The level of CoE was high, moderate, low, or very low. GRADEpro software (https://gradepro.org) was used.

## 3. Results

### 3.1. Results of the Literature Search

**Figure 1:**
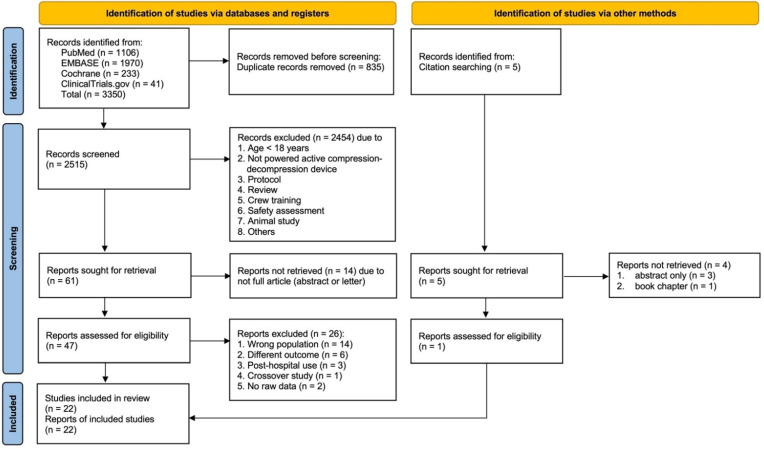
Flow diagram of preferred reporting items for systematic reviews and meta-analysis (PRISMA) 2020.

In total, 3350 articles were identified: from PubMed, 1106; EMBASE, 1970; Cochrane Library, 233; ClinicalTrials.gov, 41, and 835 articles were excluded because of duplication. A total of 2515 articles were screened by reading titles and abstracts. In total, 2454 articles were excluded that did not meet the inclusion criteria, and 61 articles were retrieved for full-text screening. Among them, 14 articles were excluded because they were not full articles. Of 47 articles assessed for eligibility, 21 articles were included, after excluding articles investigating different populations (IHCA and aircraft rescue) (*n* = 14), in-hospital use of mechanical CPR device (*n* = 3), different outcomes (*n* = 6), crossover study (*n* = 1), and no raw data for retrieval (*n* = 2). In addition, from grey literature, five articles were identified from citation searching, and one article was included in the review. In total, 22 studies were included. A flowchart according to the PRISMA statement [20] is shown in Figure 1.

Table 1 summarizes the characteristics of the included studies. Seven RCTs and 15 non-RCTs with a total of 85,975 OHCAs were included in the meta-analysis. It included studies published between 2006 and 2021, conducted in 16 countries across the continents of North America, Asia, Oceania, and Europe. In terms of automatic CPR devices, LUCAS was applied in 12 studies [18,19,26,27,28,29,30,31,32,33,34,35], AutoPulse was applied in seven studies [36,37,38,39,40,41,42], and three studies involved both devices [17,43,44].

### 3.2. Risk of Bias Assessment

For the seven enrolled RCTs including three individual RCTs and four cluster-RCTs, the overall RoB were judged as “low” in four trials [33,34,35,36], “some concerns” in two trials [19,37], and “high” in 1 trial [38] (Supplementary Table S2). The majority of RoBs arose from the domain of the randomization process. In this domain, one trial was judged as “high” because the allocation sequence concealment was not clear and with baseline difference between intervention groups [38]; two trials were judged as “some concerns” because of baseline differences between intervention groups [19,37]. From the timing of identification or recruitment of participants in a cluster-randomized trial, one cluster RCT was judged as “high,” because the participants were recruited after sending to the hospital (not before randomization of clusters) [38]. For the 15 included non-RCTs, the overall RoB were judged as “moderate” in 10 studies [17,18,26,27,29,31,39,40,41,44] and “serious” in 5 studies [28,30,32,42,43] (Supplementary Table S3). The majority of RoB arose from bias due to confounding factors (Domain 1 in ROBINS-I). However, this was inevitable because of non-randomized settings. In this case, ten non-RCTs that used appropriate analysis to control the important confounding factors were judged as “moderate” in this domain [17,18,26,27,29,31,39,40,41,44], but the other five non-RCTs did not and were judged as “serious [28,30,32,42,43]”. One study was judged as “no information” for no detailed information on the selection of participants, deviations from intended interventions, and the treatment of missing data [28].

### 3.3. Outcomes

#### 3.3.1. Primary Outcome: Return of Spontaneous Circulation

**Figure 2:**
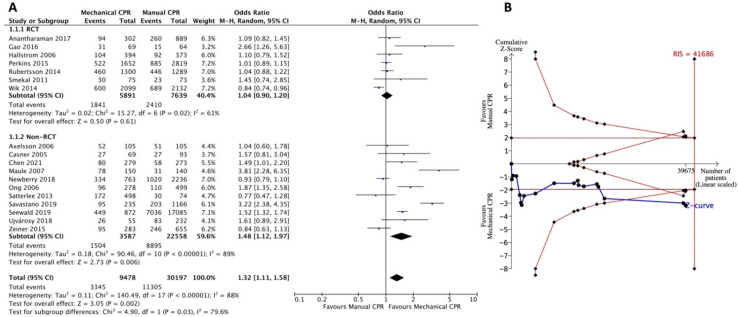
Forest plot (**A**) and trial sequential analysis (**B**) for return of spontaneous circulation between mechanical CPR device and manual CPR. CPR: cardiopulmonary resuscitation; CI: confidence interval; RCT: randomized controlled trial; RIS: required information size.

In this case, 18 of the included studies reported the outcomes of ROSC in patients with OHCA. There were 7 RCTs and 11 non-RCTs, with a total of 39,675 participants (Figure 2). The pooled estimates from both RCTs and non-RCTs revealed benefits of mechanical CPR over manual CPR in the ROSC outcome (OR = 1.32, 95% CI: 1.11–1.58). Heterogeneity among the studies was high (*I*^2^ = 88%) (Figure 2A). In the subgroup analyses of RCTs and non-RCTs, a statistical difference between the prehospital use of mechanical CPR device and manual CPR was found in the non-RCTs (OR = 1.48; 95% CI: 1.12–1.97) but not in the RCTs (OR = 1.04; 95% CI: 0.90–1.20). The heterogeneity among the RCTs and non-RCTs was high (*I*^2^ = 61% and 89%, respectively) (Figure 2A). We performed TSA to examine the results. However, the cumulative Z-curve crossed the O’Brien-Fleming boundaries before the RIS (41686 participants for required power) was reached (Figure 2B). A true-positive result indicated that the cumulative power from the available literature supports the association between ROSC achievement and prehospital use of mechanical CPR devices.

#### 3.3.2. Primary Outcome: Survival to Hospital Admission

**Figure 3:**
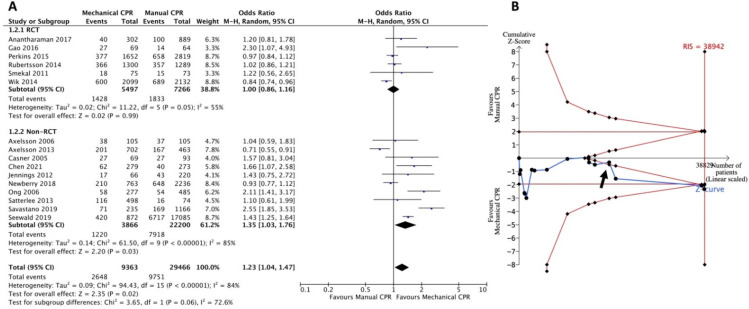
Forest plot (**A**) and trial sequential analysis (**B**) for survival to hospital admission between mechanical CPR device and manual CPR. CPR: cardiopulmonary resuscitation; CI: confidence interval; RCT: randomized controlled trial; RIS: required information size.

Six RCTs, and 10 non-RCTs reported survival to hospital admission, with a total of 38,829 patients (Figure 3A). A statistically significant difference indicated that the use of a mechanical CPR device was associated with survival to hospital admission in comparison with manual CPR (OR = 1.23, 95% CI: 1.04–1.47; *I*^2^ = 84%). The subgroup analysis showed a significant difference between the two groups in non-RCTs (OR = 1.35; 95% CI: 1.03–1.76, *I*^2^ = 85%) but not in RCTs (OR = 1.00; 95% CI: 0.86–1.16, *I*^2^ = 55%). The TSA showed that the Z-curve crossed into the futility area after the first 13 articles (Figure 3B, arrow). After enrolling the last three articles, the cumulative Z-curve finally crossed the O’Brien-Fleming boundaries before the RIS (sample size = 38942) was reached (Figure 3B). A true-positive result supported the association between achievement of survival to hospital admission and prehospital use of mechanical CPR devices.

#### 3.3.3. Secondary Outcome: Survival to Discharge

**Figure 4:**
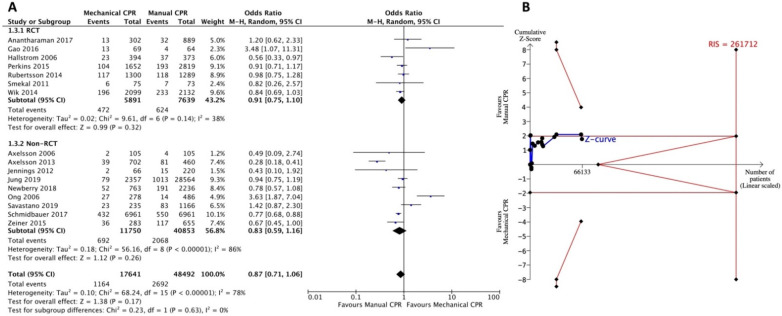
Forest plot (**A**) and trial sequential analysis (**B**) for survival to discharge between mechanical CPR device and manual CPR. CPR: cardiopulmonary resuscitation; CI: confidence interval; RCT: randomized controlled trial; RIS: required information size.

Seven RCTs and nine non-RCTs with 66,133 OHCAs were enrolled for analysis (Figure 4A). No significant benefit for survival to discharge was found when applying the mechanical CPR device compared to manual CPR (OR = 0.87; 95% CI: 0.87–1.06). There was high heterogeneity among the enrolled studies (*I*^2^ = 78%). The subgroup analysis revealed consistent results in both RCTs (OR = 0.91; 95% CI: 0.75–1.10, *I*^2^ = 38%) and non-RCTs (OR = 0.83; 95% CI: 0.59–1.16, *I*^2^ = 86%). TSA assessment showed that the RIS of 261,712 participants could not be acquired from the pooled studies (Figure 4B). In addition, the accumulative Z-curve neither crossed the O’Brien-Fleming monitoring boundary nor entered the inner border of the futility boundary. An inconclusive result was indicated.

#### 3.3.4. Secondary Outcome: Survival to Discharge with Favorable Neurologic Status

**Figure 5:**
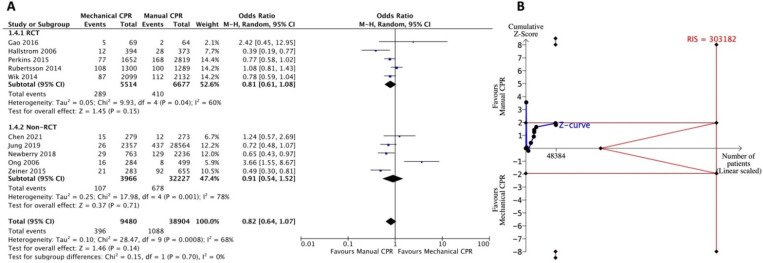
Forest plot (**A**) and trial sequential analysis (**B**) for survival to discharge with favorable neurologic status between mechanical CPR device and manual CPR. CPR: cardiopulmonary resuscitation; CI: confidence interval; RCT: randomized controlled trial; RIS: required information size.

The pooled results did not show a significant difference for discharge with favorable neurologic status between the use of mechanical CPR device and manual CPR. The pooled OR was 0.82 (95% CI: 0.64–1.07) (Figure 5A). The heterogeneity was high (*I*^2^ = 68%). Subgroup analysis showed similar results in both RCTs (OR = 0.81; 95% CI: 0.61–1.08; *I*^2^ = 60%) and non-RCTs (OR = 0.91; 95% CI: 0.54–1.52; *I*^2^ = 78%). The estimated RIS was 303,182 in TSA (Figure 5B). The Z-curve showed similar trends as that for survival to discharge, indicating an inconclusive result.

### 3.4. Subgroup and Sensitivity Analysis

Subgroup analysis of primary outcomes found that mechanical CPR devices were significantly associated with achievement of ROSC in European studies. Mechanical CPR device was significantly associated with achievement of survival to hospital admission in Asian studies. In secondary outcomes, the subgroups that were significantly associated with lower OR in achieving survival to discharge were LUCAS and the location subgroup of Europe (Table 2). Sensitivity analyses of primary and secondary outcomes are shown in Supplementary Table S4. In terms of ROSC, sensitivity analyses of fixed-effect model, non-RCTs, and studies with low RoB showed findings consistent with those of our primary analysis. In terms of survival to hospital admission, analyses of fixed-effect model and non-RCTs showed results similar to those of the primary analysis.

### 3.5. GRADE Assessment

The GRADE assessment demonstrated an overall very low CoE in the four survival outcomes (Table 3). We downgraded the overall CoE in the RoB, inconsistency, and imprecision domains. In the overall RoB, we judged as “very serious” the four outcomes because non-RCTs were enrolled with a “Moderate” overall RoB. We rated down the CoE in the domain of inconsistency in the four outcomes because high heterogeneity was consistently found. We did not rate down the CoE in the domain of indirectness because each enrolled study faced the same direction in each endpoint and compared mechanical CPR and manual CPR directly. We downgraded the domain of imprecision in the outcomes of survival to discharge and survival to discharge with favorable neurologic status because of inconclusive results and insufficient sample size in TSA. Publication bias was not observed for all the endpoints (Supplementary Figure S1).

## 4. Discussion

To date, 10 systematic reviews have been published to compare the effects of manual CPR and mechanical CPR on cardiac arrest [8,9,10,11,12,45,46,47,48,49]. Among them, five systematic reviews focused on OHCA [8,10,11,45,48], four systematic reviews, including one Cochrane review, enrolled both OHCA and IHCA [9,12,46,49], and one review involved in IHCA [47]. Among the five systematic reviews involving pure OHCA, only one systematic review (in 2011) focused on the prehospital use of mechanical CPR, suggesting that there was insufficient evidence to support mechanical CPR device use in OHCA in prehospital settings [48]. Considering the differences in medical support, etiology of cardiac arrest, survival probability between OHCA and IHCA, and diversity in the environment and medical and personnel resources between prehospital and in-hospital settings, a new SRMA is needed to provide updated evidence for the effects of mechanical CPR devices for adult OHCA in prehospital settings. In this SRMA with 85,975 OHCAs from seven RCTs and 15 non-RCTs, we first applied TSA and GRADE assessments which have not been assessed in previous SRMA. We found that mechanical CPR use in prehospital settings had higher odds of achieving ROSC (OR = 1.32; 95% CI: 1.11–1.58) and survival to hospital admission (OR = 1.23; 95% CI: 1.04–1.47) than manual CPR. TSA showed that although the RIS was not reached, there was a true statistical significance. However, because of the inclusion of non-RCTs and the inconsistency between studies, the overall CoE was very low. Moreover, according to the current evidence, we did not have enough power to assess the outcomes of survival to discharge and discharge with favorable neurologic status.

In the prehospital setting, lack of personnel, competing resuscitation tasks, fatigue, and the challenge of continuing CPR while moving the patient to the ambulance or in a moving ambulance posed obstacles. The median CPR pause time during extrication was shorter when mechanical CPR was applied (39 s, interquartile range [IQR] 29–47 s) than manual CPR (270 s, IQR 201–387 s) [50]. Safety concerns for both the patient and the rescuer have also been explored in the delivery of manual CPR in a moving vehicle [51,52]. Moreover, acceleration forces during ambulance transport affect the quality of the manual CPR [53]. Theoretically, the application of mechanical CPR in prehospital settings should improve CPR quality. This difference compared to manual CPR is not reflected in the survival outcome of OHCA [8,45]. Since 2019, several observational studies have evaluated the resuscitative effects of mechanical and manual CPR on OHCA in prehospital settings [17,18,39,44]. Three of them reported that short-term outcomes such as ROSC or survival to admission were associated with the use of mechanical CPR [17,18,39]. These findings were not included in the previous SRMA. In our TSA (Figure 2B and Figure 3B), after accumulating recent cohort studies, we found that the cumulative Z-curve finally crossed into the O’Brien-Fleming boundaries and showed the true-positive effects of mechanical CPR. This overturns the conclusion of no benefit for short-term survival outcomes in the use of mechanical CPR in the recent SRMA [8,45]. For the outcomes of ROSC and survival to hospital admission, the requirement of adequate sample size for statistical power was not met. More studies, especially large-scale high-quality RCTs, are required for any interpretation.

For the long-term survival outcomes, survival to discharge or discharge with favorable neurologic status, although not statistically significant and with insufficient statistical power to draw conclusions, the results showed the opposite trend to the short-term outcomes (Figure 4 and Figure 5). Another study showed that the majority of OHCA patients who can survive to discharge were patients with initial shockable rhythm [54]. For patients with shockable rhythm, early defibrillation may be equal or more important than CPR. Our previous study demonstrated that the benefit of mechanical CPR to achieve ROSC was more evident in OHCA patients with non-shockable rhythm but not in shockable rhythm [18]. Savastano et al. also found that mechanical CPR devices positively affect survival to discharge for witnessed cardiac arrests with non-shockable rhythm but with a neutral effect for patients with shockable rhythm [39]. A possible explanation may be that, as found in previous RCTs, the first shock delivery was delayed when applying a mechanical CPR device. The AutoPulse Assisted Prehospital International Resuscitation trial conducted by Hallstrom et al. found the mean time to first shock in ventricular fibrillation was prolonged by 2.1 min in the mechanical CPR group [37]. In the LUCAS in Cardiac Arrest (LINC) trial and the Circulation Improving Resuscitation Care (CIRC) trial, the delay in first shock delivery was 1–1.5 min longer with the device than with manual CPR [34,36]. In most RCTs, mechanical CPR administration was performed prior to cardiac rhythm assessment or delivery of the first shock [19,34,35,36,37]. Whether the delay of the first shock affects the survival outcome of OHCA with shockable rhythm needs to be further explored. However, selective use of mechanical CPR devices for patients with non-shockable rhythm or performing manual CPR first, and then switching to mechanical CPR after delivering the first shock may be a direction for further research.

In the subgroup analysis, we found that heterogeneity comes from the different study designs, and the survival benefit from mechanical CPR on short-term outcomes was from non-RCTs. There were several reasons for the synthesis of RCTs and non-RCTs for analysis. First, non-specific description of randomization sequence was noticed, and the baseline difference between groups could be identified in most cluster-RCTs [19,37,38]. Second, in individual RCTs, the randomization process was carried out once cardiac arrest was identified at the scene by the rescuers. The delay in applying mechanical CPR device may be present during the randomization and may influence the survival outcome in patients who were allocated to the mechanical CPR group [33,34,36]. Third, it is impossible for rescuers to blind the methods of CPR. Fourth, for the included non-RCTs, except for the unavoidable confounding bias, almost all considered observational studies were of high quality with low RoB (Supplementary Table S3). The above findings make the RCTs and non-RCTs comparable. Moreover, the statistical power was inadequate if only the RCTs were enrolled. Hence, we merged the evidence from both RCTs and non-RCTs and conducted a subgroup analysis according to the different study designs. Moreover, in the GRADE assessment, we downgraded the RoB and presented the faithful CoE accordingly (Table 3) [55].

This SRMA has several limitations. First, as mentioned for sufficient statistical power, we had to synthesize RCTs and non-RCTs for analysis. This also occurred in the previous SRMA. Instead, we downgraded the RoB in the GRADE assessment to carefully interpret the findings. Second, there was noticeable heterogeneity. However, the heterogeneity could be partially explained by the different study designs. There was still unobservable between-study heterogeneity, especially in terms of long-term survival outcomes. Third, post-arrest care is associated with long-term outcome [54,56]. However, the lack of post-arrest management characteristics in most studies precluded further analysis. Fourth, our meta-analysis did not demonstrate an association between complications and different manners of CPR. A network meta-analysis by Khan et al. in 2018 showed that, compared with mechanical CPR, manual CPR led to less pneumothorax and hematoma [46]. Our study cannot assess the impact of CPR-related complications on survival outcomes.

## 5. Conclusions

This SRMA suggests that prehospital use of mechanical CPR devices may benefit adult OHCA patients to achieve ROSC and survival to hospital admission. However, long-term outcomes such as survival to discharge or discharge with favorable neurological status remain inconclusive. Our finding provides the evidence and echoes the recommendations in the latest guideline of adult advanced life support, which suggest use of mechanical CPR device when high-quality manual CPR is not practical or compromises provider safety, such as during transportation to hospital in an ambulance [57]. Owing to the between-study heterogeneity and the evidence that mainly came from non-RCTs, it is necessary to conduct large-scale, high-quality randomized studies and investigate the different effects of mechanical CPR on OHCA with shockable and non-shockable rhythms.

## Figures and Tables

**Table 1:** Summary of the included studies.

Author	Year of Publication	Country	N	Study Period	Definite Study Design	Nationwide Study	Type of Mechanical Device	CPR Guideline	Witnessed Arrest	Shockable Rhythm
**Randomized studies**										
Anantharaman	2017	Singapore	1191	2011–2012	Cluster RCT	No	LUCAS	2010 ILCOR	52% man, 62% mech	17% man, 23% mech
Gao	2016	China	133	2011–2012	Cluster RCT	No	Autopulse	2010 AHA	59% man, 67% mech	13% man, 13% mech
Hallstrom	2006	USA and Canada	767	2004–2005	Cluster RCT	No	Autopulse	2000 AHA	49% man, 44% mech	32% man, 31% mech
Perkins	2015	UK	4471	2010–2013	Cluster RCT	No	LUCAS	2005/2010 ERC	62% man, 61% mech	22% man, 23% mech
Rubertsson	2014	Sweden, UK, The Netherlands	2589	2008–2013	Individually RCT	No	LUCAS	2005 ERC	72% man, 73% mech	30% man, 29% mech
Smekal	2011	Sweden	148	2005–2007	Individually RCT	No	LUCAS	2000 ERC	74% man, 68% mech	27% man, 27% mech
Wik	2014	Norway	4231	2009–2011	Individually RCT	No	Autopulse	2005 ERC/AHA	48% man, 47% mech	24% man, 21% mech
**Non-randomized studies**										
Axelsson	2006	Sweden	210	2003–2005	Prospective cohort	No	LUCAS	2000 AHA	100% man, 100% mech	32% man, 30% mech
Axelsson	2013	Sweden	1165	2007–2011	Retrospective cohort	No	LUCAS	Not reported	72%man, 73% mech	25% man, 26% mech
Casner	2005	USA	162	2003	Retrospective cohort	No	Autopulse	Not reported	Not reported	28% man, 33% mech
Chen	2021	Taiwan	552	2018–2020	Retrospective cohort	No	LUCAS	2015 AHA	53% man, 48% mech	21% man, 26% mech
Jennings	2012	Australia	286	2006–2010	Retrospective cohort	No	Autopulse	Not reported	72% man, 71% mech	36% man, 30% mech
Jung	2019	Korea	30,921	2016–2017	Prospective cohort	Yes	LUCAS/Autopulse	Not reported	47% man, 47% mech	14% man, 14% mech
Maule	2007	België	290	2004–2006	Retrospective cohort	No	LUCAS	Not reported	Not reported	Not reported
Newberry	2018	USA	2999	2013–2015	Retrospective cohort	No	LUCAS	Not reported	43% man, 37% mech	14% man, 12% mech
Ong	2006	USA	783	2001–2005	Prospective cohort	No	Autopulse	Not reported	47% man, 52% mech	20% man, 23% mech
Satterlee	2013	USA	572	2008–2010	Retrospective cohort	No	LUCAS	Not reported	61% man, 53% mech	18% man, 21% mech
Savastano	2019	Italy	1401	2015–2017	Prospective cohort	No	Autopulse	Not reported	70% man, 86% mech	14% man, 43% mech
Schmidbauer	2017	Sweden	13,922	2011–2015	Prospective cohort	Yes	LUCAS	2010 ERC	66% man, 67% mech	22% man, 23% mech
Seewald	2019	Germany	17,957	2007–2014	Retrospective cohort	Yes	LUCAS/Autopulse	Not reported	56% man, 62% mech	25% man, 33% mech
Ujvárosy	2018	Hungary	287	2010–2013	Retrospective cohort	No	LUCAS	Not reported	Not reported	Not reported
Zeiner	2015	Austria	938	2013–2014	Prospective cohort	No	LUCAS/Autopulse	Not reported	54% man, 56% mech	22% man, 34% mech

RCT: randomised controlled trial; AHA: American Heart Association; ERC: European Resuscitation Council; ILCOR: International Liaison Committee on Resuscitation Guidelines, man: manual; mech: mechanical; CPR: cardiopulmonary resuscitation.

**Table 2:** Subgroup analyses of pooled odds ratios of primary and secondary survival outcomes of OHCA.

	ROSC				Survival to Hospital Admission				Survival to Discharge				Survival to Discharge with Favorable Neurologic Status			
Subgroups	No. of Studies	Pooled OR (95% CI)	*p*	*I*^2^ (%)	No. of Studies	Pooled OR (95% CI)	*p*	*I*^2^ (%)	No. of Studies	Pooled OR (95% CI)	*p*	*I*^2^ (%)	No. of Studies	Pooled OR (95% CI)	*p*	*I*^2^ (%)
**Type of mechanical CPR device**																
LUCAS	10	1.19 (0.99–1.43)	0.06	74%	9	1.00 (0.88–1.13)	0.94	43%	8	0.74 (0.57–0.97)	0.03	78%	4	0.86 (0.67–1.12)	0.27	48%
Autopulse	6	1.65 (0.97–2.79)	0.06	94%	6	1.67 (0.97–2.84)	0.06	91%	6	1.24 (0.71–2.18)	0.45	83%	4	1.13 (0.47–2.73)	0.79	83%
LUCAS + Autopulse	2	1.15 (0.64–2.04)	0.65	92%	1	1.43 (1.25–1.64)	<0.001	NA	2	0.83 (0.60–1.15)	0.26	52%	2	0.61 (0.42–0.88)	0.009	27%
**Geographic location**																
Europe	10	1.37 (1.06–1.78)	0.02	92%	8	1.12 (0.88–1.42)	0.36	90%	9	0.77 (0.61–0.97)	0.02	79%	4	0.79 (0.61–1.02)	0.07	62%
North America	5	1.16 (0.85–1.60)	0.34	76%	4	1.33 (0.84–2.11)	0.22	79%	3	1.13 (0.45–2.84)	0.79	90%	3	0.93 (0.32–2.73)	0.89	88%
Asia	3	1.46 (0.97–2.20)	0.07	63%	3	1.51 (1.10–2.09)	0.01	23%	3	1.26 (0.72–2.18)	0.42	59%	3	0.95 (0.55–1.64)	0.86	35%
Oceania	0	NA	NA	NA	1	1.43 (0.75–2.72)	0.28	NA	1	0.43 (0.10–1.92)	0.27	NA	0	NA	NA	NA

CPR: cardiopulmonary resuscitation, OR: odds ratio, OHCA: out-of-hospital cardiac arrest, NA: not applicable, ROSC: return of spontaneous circulation.

**Table 3:** GRADE assessment.

Mechanical CPR Compared to Manual CPR for Out-of-Hospital Cardiac Arrest							
Certainty Assessment							Summary of Findings
No. of Participants (Studies)	Risk of Bias	Inconsistency	Indirectness	Imprecision	Publication Bias	Overall Certainty of Evidence	Anticipated Absolute Effects Risk Difference
**ROSC**							
39,675	very serious ^a^	serious ^b^	not serious	not serious	none	⨁◯◯◯	67 more per 1000
(7 RCTs, 11 non-RCTs)						Very Low	(from 25 more to 112 more)
**Survival to hospital admission**							
38,829	very serious ^a^	serious ^b^	not serious	not serious	none	⨁◯◯◯	47 more per 1000
(6 RCTs, 10 non-RCTs)						Very Low	(from 9 fewer to 90 more)
**Survival to discharge**							
66,133	very serious ^a^	serious ^b^	not serious	serious ^c^	none	⨁◯◯◯	7 fewer per 1000
(7 RCTs, 9 non-RCTs)						Very Low	(from 15 fewer to 3 more)
**Survival to discharge with favorable neurologic status**							
48,384	very serious ^a^	serious ^b^	not serious	serious ^c^	none	⨁◯◯◯	5 fewer per 1000
(5 RCTs, 5 non-RCTs)						Very Low	(from 10 fewer to 2 more)

^a^ Non-RCTs enrolled with moderate overall risk of bias and RCTs enrolled with some concern overall risk of bias. ^b^ High heterogeneity (*I*^2^ > 50%) between studies was found. ^c^ Insufficient sample size or inconclusive result, analyzed by trial sequential analysis. CPR: cardiopulmonary resuscitation; ROSC: return of spontaneous circulation; ED: emergency department.

## Data Availability

Data are available from the corresponding authors upon reasonable request.

## Supplementary Materials

The following supporting information can be downloaded at: https://www.mdpi.com/article/10.3390/jcm11051448/s1, Supplementary Table S1: The used search term in the databases; Supplementary Table S2: Summary of the Cochrane risk-of-bias tool for randomized trials (RoB 2.0); Supplementary Table S3: Summary of the risk of bias in non-randomized studies—of interventions (ROBINS-I) assessment; Supplementary Table S4: Sensitivity analyses of pooled odds ratios of primary and secondary survival outcomes of OHCA; Supplementary Figure S1: Funnel plots of (A) Return of spontaneous circulation, (B) Survival to hospital admission, (C) Survival to discharge, and (D) Survival to discharge with favorable neurologic status

## References

[1] Kudenchuk P.J., Sandroni C., Drinhaus H.R., Böttiger B.W., Cariou A., Sunde K., Dworschak M., Taccone F.S., Deye N., Friberg H. (2015). Breakthrough in Cardiac Arrest: Reports From the 4th Paris International Conference. Ann. Intensive Care.

[2] Kiguchi T., Okubo M., Nishiyama C., Maconochie I., Ong M.E.H., Kern K.B., Wyckoff M.H., McNally B., Christensen E.F., Tjelmeland I. (2020). Out-of-Hospital Cardiac Arrest Across the World: First Report From the International Liaison Committee on Resuscitation (ILCOR). Resuscitation.

[3] Berdowski J., Berg R.A., Tijssen J.G., Koster R.W. (2010). Global Incidences of Out-of-Hospital Cardiac Arrest and Survival Rates: Systematic Review of 67 Prospective Studies. Resuscitation.

[4] Yan S., Gan Y., Jiang N., Wang R., Chen Y., Luo Z., Zong Q., Chen S., Lv C. (2020). The Global Survival Rate Among Adult Out-of-Hospital Cardiac Arrest Patients Who Received Cardiopulmonary Resuscitation: A Systematic Review and Meta-Analysis. Crit. Care.

[5] Merchant R.M., Topjian A.A., Panchal A.R., Cheng A., Aziz K., Berg K.M., Lavonas E.J., Magid D.J., Adult Basic and Advanced Life Support, Pediatric Basic and Advanced Life Support, Neonatal Life Support, Resuscitation Education Science, and Systems of Care Writing Groups (2020). Circulation 2020-Part 1: Executive summary: 2020 American Heart Association guidelines for cardiopulmonary resuscitation and emergency cardiovascular care. Circulation.

[6] Kahn P.A., Dhruva S.S., Rhee T.G., Ross J.S. (2019). Use of Mechanical Cardiopulmonary Resuscitation Devices for Out-of-Hospital Cardiac Arrest, 2010–2016. JAMA Netw. Open..

[7] Edelson D.P., Sasson C., Chan P.S., Atkins D.L., Aziz K., Becker L.B., Berg R.A., Bradley S.M., Brooks S.C., Cheng A. (2020). Interim Guidance for Basic and Advanced Life Support in Adults, Children, and Neonates With Suspected or Confirmed COVID-19: From the Emergency Cardiovascular Care Committee and Get With the Guidelines-Resuscitation Adult and Pediatric Task Forces of the American Heart Association. Circulation.

[8] Zhu N., Chen Q., Jiang Z., Liao F., Kou B., Tang H., Zhou M. (2019). A Meta-Analysis of the Resuscitative Effects of Mechanical and Manual Chest Compression in Out-of-Hospital Cardiac Arrest Patients. Crit. Care.

[9] Wang P., Brooks S. (2020). Cochrane Corner: Are Mechanical Compressions Better Than Manual Compressions in Cardiac Arrest?. Heart.

[10] Bonnes J.L., Brouwer M.A., Navarese E.P., Verhaert D.V., Verheugt F.W., Smeets J.L., de Boer M.J. (2016). Manual Cardiopulmonary Resuscitation Versus CPR Including a Mechanical Chest Compression Device in Out-of-Hospital Cardiac Arrest: A Comprehensive Meta-Analysis From Randomized and Observational Studies. Ann. Emerg. Med..

[11] Liu M., Shuai Z., Ai J., Tang K., Liu H., Zheng J., Gou J., Lv Z. (2019). Mechanical Chest Compression With LUCAS Device Does Not Improve Clinical Outcome in Out-of-Hospital Cardiac Arrest Patients: A Systematic Review and Meta-Analysis. Medicine.

[12] Li H., Wang D., Yu Y., Zhao X., Jing X. (2016). Mechanical Versus Manual Chest Compressions for Cardiac Arrest: A Systematic Review and Meta-Analysis. Scand. J. Trauma Resusc. Emerg. Med..

[13] Hayashida K., Tagami T., Fukuda T., Suzuki M., Yonemoto N., Kondo Y., Ogasawara T., Sakurai A., Tahara Y., Nagao K. (2017). Mechanical Cardiopulmonary Resuscitation and Hospital Survival Among Adult Patients With Nontraumatic Out-of-Hospital Cardiac Arrest Attending the Emergency Department: A Prospective, Multicenter, Observational Study in Japan (SOS-KANTO [Survey of Survivors after Out-Of-Hospital Cardiac Arrest in Kanto Area] 2012 Study). J. Am. Heart Assoc..

[14] Kim H.T., Kim J.G., Jang Y.S., Kang G.H., Kim W., Choi H.Y., Jun G.S. (2019). Comparison of in-Hospital Use of Mechanical Chest Compression Devices for Out-of-Hospital Cardiac Arrest Patients: AUTOPULSE vs LUCAS. Medicine.

[15] Lin C.K., Huang M.C., Feng Y.T., Jeng W.H., Chung T.C., Lau Y.W., Cheng K.I. (2015). Effectiveness of Mechanical Chest Compression for Out-of-Hospital Cardiac Arrest Patients in an Emergency Department. J. Chin. Med. Assoc..

[16] Ogawa Y., Shiozaki T., Hirose T., Ohnishi M., Nakamori Y., Ogura H., Shimazu T. (2015). Load-Distributing-Band Cardiopulmonary Resuscitation for Out-of-Hospital Cardiac Arrest Increases Regional Cerebral Oxygenation: A Single-Center Prospective Pilot Study. Scand. J. Trauma Resusc. Emerg. Med..

[17] Seewald S., Obermaier M., Lefering R., Bohn A., Georgieff M., Muth C.M., Gräsner J.T., Masterson S., Scholz J., Wnent J. (2019). Application of Mechanical Cardiopulmonary Resuscitation Devices and Their Value in Out-of-Hospital Cardiac Arrest: A Retrospective Analysis of the German Resuscitation Registry. PLoS ONE.

[18] Chen Y.R., Liao C.J., Huang H.C., Tsai C.H., Su Y.S., Liu C.H., Hsu C.F., Tsai M.J. (2021). The Effect of Implementing Mechanical Cardiopulmonary Resuscitation Devices on Out-of-Hospital Cardiac Arrest Patients in an Urban City of Taiwan. Int. J. Environ. Res. Public Health.

[19] Anantharaman V., Ng B.L., Ang S.H., Lee C.Y., Leong S.H., Ong M.E., Chua S.J., Rabind A.C., Anjali N.B., Hao Y. (2017). Prompt Use of Mechanical Cardiopulmonary Resuscitation in Out-of-Hospital Cardiac Arrest: The MECCA Study Report. Singap. Med. J..

[20] Page M.J., McKenzie J.E., Bossuyt P.M., Boutron I., Hoffmann T.C., Mulrow C.D., Shamseer L., Tetzlaff J.M., Akl E.A., Brennan S.E. (2021). The PRISMA 2020 Statement: An Updated Guideline for Reporting Systematic Reviews. BMJ.

[21] Sterne J.A.C., Savović J., Page M.J., Elbers R.G., Blencowe N.S., Boutron I., Cates C.J., Cheng H.Y., Corbett M.S., Eldridge S.M. (2019). RoB 2: A Revised Tool for Assessing Risk of Bias in Randomised Trials. BMJ.

[22] Sterne J.A., Hernán M.A., Reeves B.C., Savović J., Berkman N.D., Viswanathan M., Henry D., Altman D.G., Ansari M.T., Boutron I. (2016). Robins-I: A Tool for Assessing Risk of Bias in Non-Randomised Studies of Interventions. BMJ.

[23] Brok J., Thorlund K., Wetterslev J., Gluud C. (2009). Apparently Conclusive Meta-Analyses May Be Inconclusive-Trial Sequential Analysis Adjustment of Random Error Risk Due to Repetitive Testing of Accumulating Data in Apparently Conclusive Neonatal Meta-Analyses. Int. J. Epidemiol..

[24] Kang H. (2021). Trial Sequential Analysis: Novel Approach for Meta-Analysis. Anesth. Pain Med..

[25] Guyatt G.H., Oxman A.D., Vist G.E., Kunz R., Falck-Ytter Y., Alonso-Coello P., Schünemann H.J., GRADE Working Group (2008). GRADE: An Emerging Consensus on Rating Quality of Evidence and Strength of Recommendations. BMJ.

[26] Axelsson C., Herrera M.J., Fredriksson M., Lindqvist J., Herlitz J. (2013). Implementation of Mechanical Chest Compression in Out-of-Hospital Cardiac Arrest in an Emergency Medical Service System. Am. J. Emerg. Med..

[27] Axelsson C., Nestin J., Svensson L., Axelsson A.B., Herlitz J. (2006). Clinical Consequences of the Introduction of Mechanical Chest Compression in the EMS System for Treatment of Out-of-Hospital Cardiac Arrest-A Pilot Study. Resuscitation.

[28] Maule Y. (2007). Mechanical External Chest Compression: A New Adjuvant Technology in Cardiopulmonary Resuscitation. Urgences Accueil..

[29] Newberry R., Redman T., Ross E., Ely R., Saidler C., Arana A., Wampler D., Miramontes D. (2018). No Benefit in Neurologic Outcomes of Survivors of Out-of-Hospital Cardiac Arrest With Mechanical Compression Device. Prehosp. Emerg. Care.

[30] Satterlee P.A., Boland L.L., Johnson P.J., Hagstrom S.G., Page D.I., Lick C.J. (2013). Implementation of a Mechanical Chest Compression Device as Standard Equipment in a Large Metropolitan Ambulance Service. J. Emerg. Med..

[31] Schmidbauer S., Herlitz J., Karlsson T., Axelsson C., Friberg H. (2017). Use of Automated Chest Compression Devices After Out-of-Hospital Cardiac Arrest in Sweden. Resuscitation.

[32] Ujvárosy D., Sebestyén V., Pataki T., Ötvös T., Lőrincz I., Paragh G., Szabó Z. (2018). Cardiovascular Risk Factors Differently Affect the Survival of Patients Undergoing Manual or Mechanical Resuscitation. BMC Cardiovasc. Disord..

[33] Smekal D., Johansson J., Huzevka T., Rubertsson S. (2011). A Pilot Study of Mechanical Chest Compressions With the LUCAS™ Device in Cardiopulmonary Resuscitation. Resuscitation.

[34] Rubertsson S., Lindgren E., Smekal D., Östlund O., Silfverstolpe J., Lichtveld R.A., Boomars R., Ahlstedt B., Skoog G., Kastberg R. (2014). Mechanical Chest Compressions and Simultaneous Defibrillation vs Conventional Cardiopulmonary Resuscitation in Out-of-Hospital Cardiac Arrest: The LINC Randomized Trial. JAMA.

[35] Perkins G.D., Lall R., Quinn T., Deakin C.D., Cooke M.W., Horton J., Lamb S.E., Slowther A.M., Woollard M., Carson A. (2015). Mechanical Versus Manual Chest Compression for Out-of-Hospital Cardiac Arrest (PARAMEDIC): A Pragmatic, Cluster Randomised Controlled Trial. Lancet.

[36] Wik L., Olsen J.A., Persse D., Sterz F., Lozano M., Brouwer M.A., Westfall M., Souders C.M., Malzer R., van Grunsven P.M. (2014). Manual vs. Integrated Automatic Load-Distributing Band CPR With Equal Survival After out of Hospital Cardiac Arrest. The Randomized CIRC Trial. Resuscitation.

[37] Hallstrom A., Rea T.D., Sayre M.R., Christenson J., Anton A.R., Mosesso V.N., Van Ottingham L., Olsufka M., Pennington S., White L.J. (2006). Manual Chest Compression vs Use of an Automated Chest Compression Device During Resuscitation Following Out-of-Hospital Cardiac Arrest: A Randomized Trial. JAMA.

[38] Gao C., Chen Y., Peng H., Chen Y., Zhuang Y., Zhou S. (2016). Clinical Evaluation of the AutoPulse Automated Chest Compression Device for Out-of-Hospital Cardiac Arrest in the Northern District of Shanghai, China. Arch. Med. Sci..

[39] Savastano S., Baldi E., Palo A., Raimondi M., Belliato M., Compagnoni S., Buratti S., Cacciatore E., Canevari F., Iotti G. (2019). Load Distributing Band Device for Mechanical Chest Compressions: An Utstein-Categories Based Analysis of Survival to Hospital Discharge. Int. J. Cardiol..

[40] Ong M.E., Ornato J.P., Edwards D.P., Dhindsa H.S., Best A.M., Ines C.S., Hickey S., Clark B., Williams D.C., Powell R.G. (2006). Use of an Automated, Load-Distributing Band Chest Compression Device for Out-of-Hospital Cardiac Arrest Resuscitation. JAMA.

[41] Jennings P.A., Harriss L., Bernard S., Bray J., Walker T., Spelman T., Smith K., Cameron P. (2012). An Automated CPR Device Compared With Standard Chest Compressions for Out-of-Hospital Resuscitation. BMC Emerg. Med..

[42] Casner M., Andersen D., Isaacs S.M. (2005). The Impact of a New CPR Assist Device on Rate of Return of Spontaneous Circulation in Out-of-Hospital Cardiac Arrest. Prehosp. Emerg. Care.

[43] Zeiner S., Sulzgruber P., Datler P., Keferböck M., Poppe M., Lobmeyr E., van Tulder R., Zajicek A., Buchinger A., Polz K. (2015). Mechanical Chest Compression Does Not Seem to Improve Outcome After Out-Of Hospital Cardiac Arrest. A Single Center Observational Trial. Resuscitation.

[44] Jung E., Park J.H., Lee S.Y., Ro Y.S., Hong K.J., Song K.J., Ryu H.H., Shin S.D. (2020). Mechanical Chest Compression Device for Out-of-Hospital Cardiac Arrest: A Nationwide Observational Study. J. Emerg. Med..

[45] Sheraton M., Columbus J., Surani S., Chopra R., Kashyap R. (2021). Effectiveness of Mechanical Chest Compression Devices Over Manual Cardiopulmonary Resuscitation: A Systematic Review With Meta-Analysis and Trial Sequential Analysis. West. J. Emerg. Med..

[46] Khan S.U., Lone A.N., Talluri S., Khan M.Z., Khan M.U., Kaluski E. (2018). Efficacy and Safety of Mechanical Versus Manual Compression in Cardiac Arrest—A Bayesian Network Meta-Analysis. Resuscitation.

[47] Couper K., Yeung J., Nicholson T., Quinn T., Lall R., Perkins G.D. (2016). Mechanical Chest Compression Devices at in-Hospital Cardiac Arrest: A Systematic Review and Meta-Analysis. Resuscitation.

[48] Ong M.E., Mackey K.E., Zhang Z.C., Tanaka H., Ma M.H., Swor R., Shin S.D. (2012). Mechanical CPR Devices Compared to Manual CPR During Out-of-Hospital Cardiac Arrest and Ambulance Transport: A Systematic Review. Scand. J. Trauma Resusc. Emerg. Med..

[49] Wang P.L., Brooks S.C. (2018). Mechanical Versus Manual Chest Compressions for Cardiac Arrest. Cochrane Database Syst. Rev..

[50] Lyon R.M., Crawford A., Crookston C., Short S., Clegg G.R. (2015). The Combined Use of Mechanical CPR and a Carry Sheet to Maintain Quality Resuscitation in Out-of-Hospital Cardiac Arrest Patients During Extrication and Transport. Resuscitation.

[51] Becker L.R., Zaloshnja E., Levick N., Li G., Miller T.R. (2003). Relative Risk of Injury and Death in Ambulances and Other Emergency Vehicles. Accid. Anal. Prev..

[52] Kahn C.A., Pirrallo R.G., Kuhn E.M. (2001). Characteristics of Fatal Ambulance Crashes in the United States: An 11-Year Retrospective Analysis. Prehosp. Emerg. Care.

[53] Kurz M.C., Dante S.A., Puckett B.J. (2012). Estimating the Impact of Off-Balancing Forces Upon Cardiopulmonary Resuscitation During Ambulance Transport. Resuscitation.

[54] Hassager C., Nagao K., Hildick-Smith D. (2018). Out-of-Hospital Cardiac Arrest: In-Hospital Intervention Strategies. Lancet.

[55] Schünemann H.J., Cuello C., Akl E.A., Mustafa R.A., Meerpohl J.J., Thayer K., Morgan R.L., Gartlehner G., Kunz R., Katikireddi S.V. (2019). [GRADE guidelines, 105–114] [GRADE guidelines, 105–114]. GRADE Guidelines: 18. How Robins-I and Other Tools to Assess Risk of Bias in Nonrandomized Studies Should Be Used to Rate the Certainty of a Body of Evidence. J. Clin. Epidemiol..

[56] Chang H.C., Tsai M.S., Kuo L.K., Hsu H.H., Huang W.C., Lai C.H., Shih M.C., Huang C.H. (2022). Factors Affecting Outcomes in Patients With Cardiac Arrest Who Receive Target Temperature Management: The Multi-Center TIMECARD Registry. J. Formos. Med. Assoc..

[57] Soar J., Böttiger B.W., Carli P., Couper K., Deakin C.D., Djärv T., Lott C., Olasveengen T., Paal P., Pellis T. (2021). European Resuscitation Council Guidelines 2021: Adult Advanced Life Support. Resuscitation.

