# Supplementary material for: Comparison between Prehospital Mechanical Cardiopulmonary Resuscitation (CPR) Devices and Manual CPR for Out-of-Hospital Cardiac Arrest: A Systematic Review, Meta-Analysis, and Trial Sequential Analysis
Source: J Clin Med. 2022 Mar 7;11(5):1448. doi: 10.3390/jcm11051448 (PMC8911115; doi:10.3390/jcm11051448)

Supplementary Table S1. The used search term in the databases

Library	Search term	Restriction
Pubmed	("Heart arrest"[All Fields] OR "cardiac arrest"[All Fields]) AND ("cardiopulmonary resuscitation"[All Fields] OR "CPR"[All Fields] OR "chest compression"[All Fields]) AND ("Mechanical"[All Fields] OR "LUCAS"[All Fields] OR "AutoPulse"[All Fields] OR "Load distributing band"[All Fields])	All Fields
Cochrane Library	("Heart arrest" OR "cardiac arrest") AND ("cardiopulmonary resuscitation" OR "CPR" OR "chest compression") AND ("Mechanical" OR "LUCAS" OR "AutoPulse" OR "Load distributing band") (Word variations have been searched)	All Text
Embase	#1. 'heart arrest' OR 'cardiac arrest'<br>#2. 'cpr' OR 'cardiopulmonary resuscitation' OR 'chest compression'<br>#3. 'lucas' OR 'autopulse' OR 'mechanical' OR 'load distributing band'<br>#4. #1 AND #2 AND #3	No
ClinicalTrials.gov	"Heart arrest" OR "Cardiac arrest" | ("Cardiopulmonary resuscitation" OR "CPR" OR "Chest compression") AND ("LUCAS" OR "Autopulse" OR "Load distributing band" OR "Mechanical")	No

Supplementary Table S2. Summary of the Cochrane risk-of-bias tool for randomised trials (RoB 2.0)

Type of RCT	Study	D1	D1b	D2	D3	D4	D5	Overall
Cluster RCT	Anantharaman 2017	Some concerns	Low	Low	Low	Low	Low	Some concerns
	Gao 2016	High	High	Low	Low	Low	Low	High
	Hallstrom 2006	Some concerns	Low	Low	Low	Low	Low	Some concerns
	Perkins 2015	Low	Low	Low	Low	Low	Low	Low
Individually RCT	Rubertsson 2014	Low		Low	Low	Low	Low	Low
	Smekal 2011	Low		Low	Low	Low	Low	Low
	Wik 2014	Low		Low	Low	Low	Low	Low

D1: Risk of bias arising from the randomisation process.

D1b: Risk of bias arising from the timing of identification or recruitment of participants in a cluster-randomised trial.

D2: Risk of bias due to deviations from the intended interventions.

D3: Risk of bias due to missing outcome data.

D4: Risk of bias in measurement of the outcome

D5: Risk of bias in selection of the reported result

RCT: Randomised controlled trial

Supplementary Table S3. Summary of The Risk Of Bias In Non-randomised Studies – of Interventions (ROBINS-I) assessment

Study	D1	D2	D3	D4	D5	D6	D7	Overall
Axelsson 2006	Moderate	Low	Low	Low	Low	Low	Low	Moderate
Axelsson 2013	Moderate	Low	Low	Low	Low	Low	Low	Moderate
Casner 2005	Serious	Low	Low	Low	Low	Low	Low	Serious
Chen 2021	Moderate	Low	Low	Low	Low	Low	Low	Moderate
Jennings 2012	Moderate	Low	Low	Low	Low	Low	Low	Moderate
Jung 2019	Moderate	Low	Low	Low	Low	Low	Low	Moderate
Maule 2007	Serious	No information	Low	No information	No information	Low	Low	Serious
Newberry 2018	Moderate	Low	Low	Low	Low	Low	Low	Moderate
Ong 2006	Moderate	Low	Low	Low	Low	Low	Low	Moderate
Satterlee 2013	Serious	Low	Low	Low	Low	Low	Low	Serious
Savastano 2019	Moderate	Low	Low	Low	Low	Low	Low	Moderate
Schmidbauer 2017	Moderate	Low	Low	Low	Low	Low	Low	Moderate
Seewald 2019	Moderate	Low	Low	Low	Low	Low	Low	Moderate
Ujvárosy 2018	Serious	Low	Low	Low	Low	Low	Low	Serious
Zeiner 2015	Serious	Low	Low	Low	Low	Low	Low	Serious

D1: Bias due to confounding

D2: Bias in selection of participants into the study

D3: Bias in classification of interventions

D4: Bias due to deviations from intended interventions

D5: Bias due to missing data

D6: Bias in measurement of outcomes

D7: Bias in selection of the reported result

Supplementary Table S4. Sensitivity analyses of pooled odds ratios of primary and secondary survival outcomes of OHCA

	ROSC				Survival to hospital admission				Survival to discharge				Survival to discharge with favourable neurologic status			
	No. of Studies	Pooled OR (95% CI)	*P*	I^2 (%)	No. of Studies	Pooled OR (95% CI)	*P*	I^2 (%)	No. of Studies	Pooled OR (95% CI)	*P*	I^2 (%)	No. of Studies	Pooled OR (95% CI)	*P*	I^2 (%)
**Random effect**	18	1.32 (1.11 – 1.58)	0.002	88%	16	1.23 (1.04 – 1.47)	0.02	84%	16	0.87 (0.71 – 1.06)	0.17	78%	10	0.82 (0.64 – 1.07)	0.14	68%
**Fixed effect**	18	1.13 (1.07 – 1.20)	<0.001	88%	16	1.08 (1.02 – 1.14)	0.01	84%	16	0.82 (0.76 – 0.89)	<0.001	78%	10	0.80 (0.71 – 0.91)	<0.001	68%
**Study design**																
RCT	7	1.04 (0.90 – 1.20)	0.61	61%	6	1.00 (0.86 – 1.16)	0.99	55%	7	0.91 (0.75 – 1.10)	0.32	38%	5	0.81 (0.61 – 1.08)	0.15	60%
non-RCT	11	1.48 (1.12 – 1.97)	0.006	89%	10	1.35 (1.03 – 1.76)	0.03	85%	9	0.83 (0.59 – 1.16)	0.26	86%	5	0.91 (0.54 – 1.52)	0.71	78%
**Low risk of bias***	10	1.30 (1.04 – 1.63)	0.02	91%	12	1.20 (0.99 – 1.46)	0.07	88%	12	0.87 (0.69 – 1.08)	0.21	81%	7	0.91 (0.70 – 1.18)	0.47	66%

RCT: randomized-control trials, OR: odds ratio, OHCA: out-of-hospital cardiac arrest, ROSC: return of spontaneous circulation

*Low risk of bias studies included RCTs with low overall risk of bias evaluated by ROB2.0 and non-RCTs with moderate risk of bias evaluated by ROBINS-1

Supplementary Figure S1. Funnel plots of (A) Return of spontaneous circulation, (B) Survival to hospital admission, (C) Survival to discharge, and (D) Survival to discharge with favorable neurologic status. OR, odds ratio; RCT, randomised controlled trial; Non-RCT, non-randomised controlled trial.

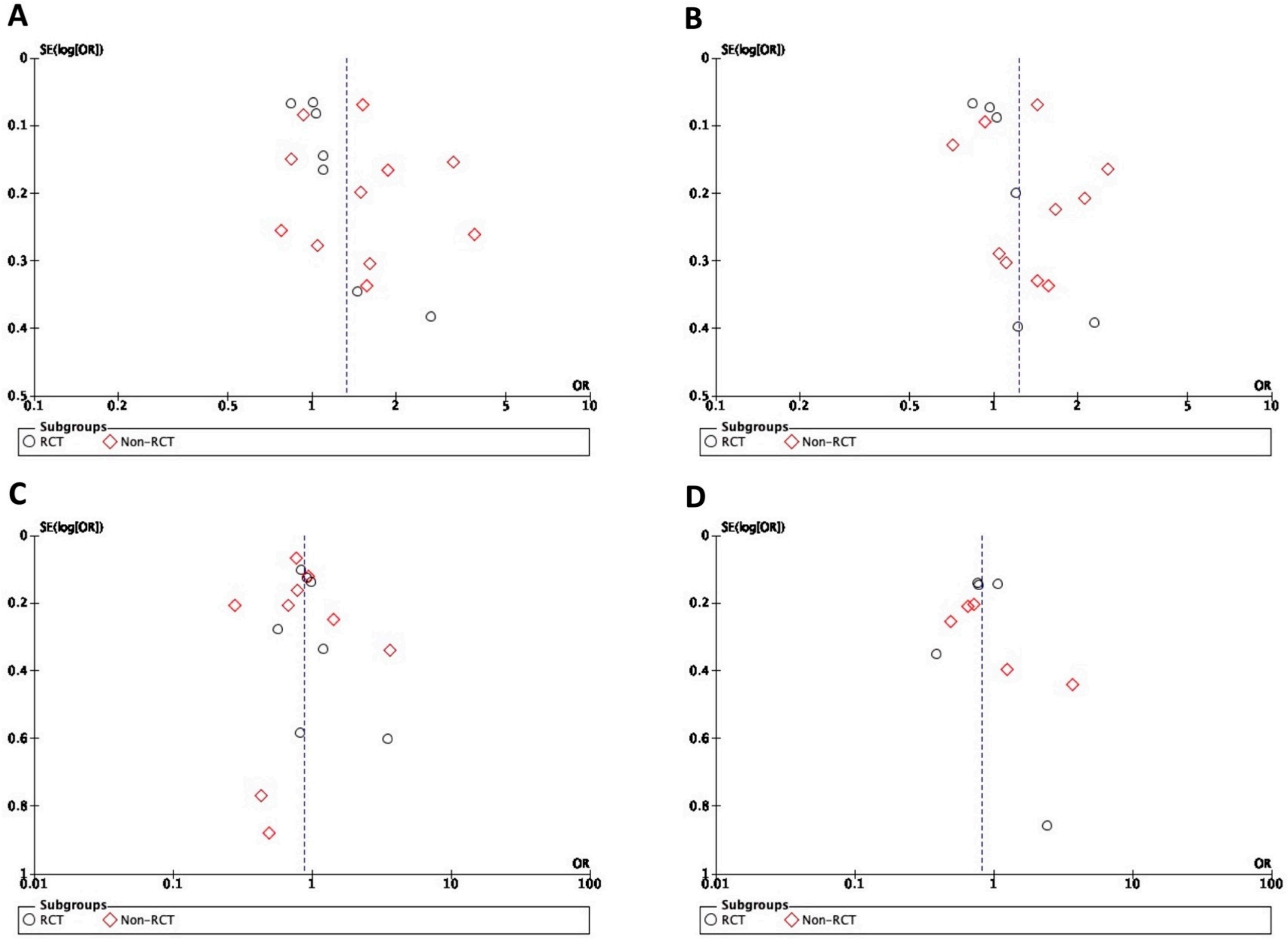

Supplement: Supplementary file 1 [file jcm-11-01448-s001.zip › jcm-1602025-supplementary.pdf]
